# Establishing a New Link between Fuzzy Logic, Neuroscience, and Quantum Mechanics through Bayesian Probability: Perspectives in Artificial Intelligence and Unconventional Computing

**DOI:** 10.3390/molecules26195987

**Published:** 2021-10-02

**Authors:** Pier Luigi Gentili

**Affiliations:** Department of Chemistry, Biology, and Biotechnology, Università degli Studi di Perugia, Via Elce di sotto 8, 06123 Perugia, Italy; pierluigi.gentili@unipg.it; Tel.: +39-0755855573

**Keywords:** uncertainty, fuzzy set, brain, neocortex, cortical columns, QBism, wavefunctions, maximum entropy method, responsive molecules, smart materials

## Abstract

Human interaction with the world is dominated by uncertainty. Probability theory is a valuable tool to face such uncertainty. According to the Bayesian definition, probabilities are personal beliefs. Experimental evidence supports the notion that human behavior is highly consistent with Bayesian probabilistic inference in both the sensory and motor and cognitive domain. All the higher-level psychophysical functions of our brain are believed to take the activities of interconnected and distributed networks of neurons in the neocortex as their physiological substrate. Neurons in the neocortex are organized in cortical columns that behave as fuzzy sets. Fuzzy sets theory has embraced uncertainty modeling when membership functions have been reinterpreted as possibility distributions. The terms of Bayes’ formula are conceivable as fuzzy sets and Bayes’ inference becomes a fuzzy inference. According to the QBism, quantum probabilities are also Bayesian. They are logical constructs rather than physical realities. It derives that the Born rule is nothing but a kind of Quantum Law of Total Probability. Wavefunctions and measurement operators are viewed epistemically. Both of them are similar to fuzzy sets. The new link that is established between fuzzy logic, neuroscience, and quantum mechanics through Bayesian probability could spark new ideas for the development of artificial intelligence and unconventional computing.

## 1. Introduction

Scientific knowledge is expressed through mathematical equations, algorithms, and sentences formulated in natural language. It develops through inductive, deductive, and abductive reasonings mainly based on data and information collected by means of quantitative measurements and qualitative observations. The data and information which are the pillars of scientific knowledge are unavoidably tainted by uncertainty. In fact, science is said to be exact, not as it is based on data that are infinitely accurate, but as its rigorous methodology allows determining the extent of uncertainty associated with every experimental determination [1].

One relevant purpose of science is that of making predictions about natural phenomena. When we deal with macroscopic phenomena, we can distinguish three principal scenarios. First, the natural phenomena are deterministic. In this case, we can predict the evolution of the events with an accuracy that depends on the uncertainty in the definition of the initial conditions and the errors we introduce in the computation of the system’s evolution. A second scenario occurs when the phenomena are deterministic but chaotic. In this case, the solution of the differential equations describing the dynamics of the chaotic system allows one to make reasonable predictions only in the very short term. We might use fuzzy logic or alternative algorithms, such as the Artificial Neural Networks or Local Nonlinear Predictors [2,3,4] to make reasonable predictions for a more extended period of time. Finally, there is a third situation: it encompasses all those complex nonlinear phenomena that appear stochastic due to the many variables that are involved and the lack of knowledge of their ruling laws. Examples are how the insurgence of a disease depends on our lifestyle, or how a financial crisis can be triggered by political decisions. In complex situations, statistical methods based on the theory of probability are traditionally employed to make forecasts and take decisions. When the stochastic variables change more quickly than the other controlled variables, the Langevin equation can be used. In the last years, the trust in alternative algorithms, such as Fuzzy Logic Systems and Artificial Neural Networks, has been soaring. Fuzzy Logic Systems mainly relate to human expertise and the ability to compute with words in complex situations [5]. On the other hand, Artificial Neural Networks are computer-based algorithms, modeled on the structure and behavior of neurons in the human brain, which can be trained, as powerful “black boxes,” to recognize and categorize complex patterns [6].

A rigorous description of the events occurring at the molecular level is accomplished by using the laws of quantum mechanics. Quantum mechanics can be thought of as the theory of constructing wavefunctions. A wavefunction encodes the properties of a particular quantum system and the essential details of the specific experiment that is performed on that system. It is possible to extract predictions of measurable outcomes by solving the Schrödinger equation, i.e., determining the eigenvalues of the eigenvectors that are the wavefunctions. Unfortunately, solving the Schrödinger equation means facing what, in the field of Computational Complexity, is called an instance of an exponential problem. The number of computational steps needed to solve the Schrödinger equation grows as $2^{N}$, with N being the number of particles [7]. According to the theory of Computational Complexity, we cannot determine the exact solution of the Schrödinger equation in a reasonable lapse of time when it refers to a microscopic system with many particles, i.e., when N is large. Therefore, we must settle for approximate solutions that are achievable in a reasonable lapse of time through easily accessible computational facilities. Furthermore, in the microscopic world, the Heisenberg’s Uncertainty Principle holds. The Uncertainty Principle imposes limits in our prediction of the dynamics of a particle, as its position and momentum cannot be determined accurately and simultaneously. It derives that the description of the microscopic phenomena is approximate and uncertain. The consequence is that quantum mechanics grounds on the theory of probability. There are two schools of thought regarding the interpretation of probability: the “classical”, or “frequentist”, school and the subjectivist or “Bayesian” school. As there are two sharply distinct definitions of probability, there are also two fundamentally different interpretations of quantum mechanics: the traditional one, which is based on the frequentist definition of probability, and a more recent one, named QBism, which is based on the Bayes’ theory [8].

### Purpose of This Work

As both fuzzy logic and human reasoning are also related to Bayesian probability, the purpose of this paper is to establish a new link between QBism, fuzzy logic, and neuroscience. Fuzzy logic plays a relevant role in the field of artificial intelligence as it is a good model of human ability to compute with words [9]. Recently, it has been shown that fuzzy logic is also processed at the molecular, supramolecular, and systems levels [10]. Therefore, the conceptual relationship between fuzzy logic, neuroscience, and QBism promises to be fertile for developing new interdisciplinary ideas in the field of artificial intelligence and unconventional computing.

**Scheme 1:**
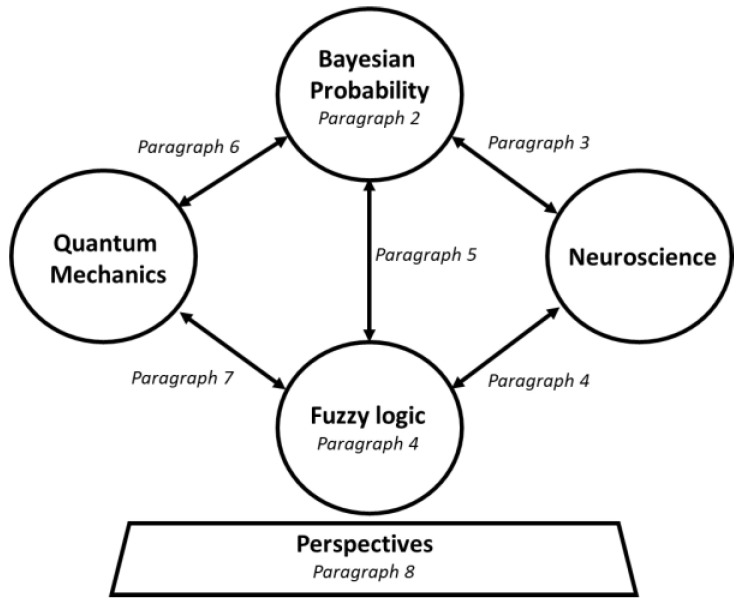
Structure of the paper.

Structure of this article (also see Scheme 1):

- The two principal definitions of classical probability, with an emphasis on the Bayes’ theory, are recalled in paragraph 2.
- The role of Bayes’ probability in neuroscience is surveyed in paragraph 3.
- The links between neuroscience and fuzzy logic are presented in paragraph 4.
- The relationship between Bayesian probability and fuzzy sets and logic is developed in paragraph 5 through the Information Principle [11].
- The interpretation of quantum mechanics according to the theory of QBism is summarized in paragraph 6. The new link that QBism allows to tie between quantum and fuzzy logic is presented in paragraph 7, after mentioning other previous bonds between the two theories.
- The perspectives of the new transdisciplinary link between fuzzy logic, neuroscience, and quantum mechanics are outlined in the last paragraph.

## 2. The Definitions of Classical Probability

**Figure 1:**
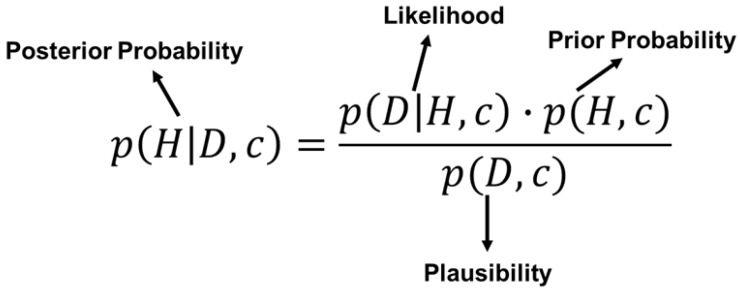
The Bayes’ formula: D represents the data we gather about a hypothesis H, whose probability we want to estimate, within the context c.

Probability has been a subject of debate among scholars for centuries. According to the frequentist definition, thoroughly exposed for the first time by John Venn (1834–1923) [12], the probability of a random event is the number of favorable cases divided by all cases. This definition is based on counting the occurrences of events. It is not always meaningful to relate uncertainty to frequency. Physicists adopted frequentism as laboratory experiments in physically isolated systems are, in principle, easily repeated many times. Observations can be repeated many times indefinitely under some standard, stable conditions. However, some types of events are rare or unrepeatable, or statistical data may simply be unavailable; these kinds of events are frequently experienced in biology, psychology, economics, medical science, and any other discipline that deals with Complex Systems [1,13]. These limitations do not prevent people from thinking that some events are more possible, probable, or certain than others. In these situations, we can rely upon the subjectivist definition of probability. According to Thomas Bayes (1701–1761), who was a presbyterian minister and an able mathematician and statistician [14], probabilities are beliefs. Probabilities do not exist [15]: they are not facts or objects, but they are malleable beliefs. The Bayes’ rule is a relationship among four different probabilities, which are all expressed as numbers between 0 and 1 (see Figure 1) [16]. If we indicate with D the data or the evidence that we have collected to estimate the probability of a hypothesis indicated by H, then the probability of H given D in the context *c* (i.e., p(H|D,c), where | means “given”) is the posterior probability that, according to the equation shown in Figure 1, depends on:

1. The prior probability, p(H,c), i.e., the probability of H before gathering the data D;

2. The likelihood, p(D|H,c), i.e., the probability of having the data D, i.e., the evidence, if we knew for sure that H is true;

3. The plausibility, p(D,c), i.e., the probability of collecting the data D. It is a normalizing constant, as p(D,c)=p(D|H,c)·p(H,c)+p(D|H,c)·p(H,c) [17].

Generally, it is easier to determine the probability that a particular outcome would occur given a model or hypothesis H than the other way around. Bayes’ theorem allows the probability of the model given the data to be inferred from the corresponding likelihood and prior [17]. Bayesian inference is an updating process as the (prior) probability of the hypothesis is combined with the likelihood (based on the data D) to give the new (posterior) probability of the hypothesis. The posterior probability can become a new prior probability when Bayes’ formula is reused to compute a new posterior given new data or evidence. If the new data are irrelevant to the proposition of interest, the posterior probability maintains equal to the prior. When all probabilities are either 0 or 1, probability reduces to binary logic [17]. However, most, if not all, of the real-world propositions are not known to this degree of certainty. Therefore, the usual trick is to pretend that a probability very close to either 0 or 1 can be treated as if it is 0 or 1, allowing logical reasoning to replace probabilistic reasoning.

## 3. The Bayesian Probability and Neuroscience

The Bayes’ theory asserts that probability is not an attribute of the random events of the external world, but it depends on a person’s mind, called an agent. Bayesian probability includes an agent’s personal degree of belief that an event will occur, or that a proposition is true. Personal degrees of belief may change, and therefore probability assignments for events change as well [14]. There is strong experimental evidence in support of the notion that human behavior is highly consistent with Bayesian probabilistic inference in both the sensory and motor, and cognitive domain [18]. Within our brain, information is encoded as spatiotemporal patterns of activity of cortical neurons. All the higher-level psychophysical functions, such as sensory perception, object- and event-representation, planning, and decision making, are believed to take the activities of interconnected and distributed networks of neurons in the neocortex as their physiological substrate [19]. The neocortex is that part of the brain which makes up the outer 2 to 4 mm of the two cerebral hemispheres. It is the so-called “gray-matter” that lies atop the cerebral “white-matter” composed of myelinated axons that interconnect different regions of the brain. The neocortex contains up to 28 × 10^9^ neurons and approximately the same number of glial cells. Cortical neurons are connected with each other and with neurons of other parts of the brain by a vast number of synapses of the order of $10^{12}$ [20]. The neocortex is usually described as being arranged horizontally in six laminae and vertically in a hive or mosaic of cylindrical patches with a diameter varying from 300 to 600 μm and a height spanning the entire depth of the neocortex. These cylinders, which are often considered the basic functional units of the neocortex, are called cortical columns. Neurons belonging to different cortical columns may be linked synaptically across the horizontal laminae [20]. The columnar organization allows for intermittently recursive mapping so that variables can be mapped to the two-dimensional surface of the neocortex and across the horizontal laminae. If the symbol CC stands for cortical columns, and E stands for either an image of the world in a sensory process or a resolution in the cognitive domain or a goal in motor control [21] (for example, reaching an object at a particular location) in the context c, the Bayes’ rule becomes:

(1) $$p\left(E|CC,c\right)=\frac{p\left(CC|E,c\right)\cdot p\left(E,c\right)}{p\left(CC,c\right)}$$

The posterior distribution p(E|CC,c) represents the probability of E given the activity of specific CC in the context c. It is obtained by multiplying the likelihood function, p(CC|E,c), with the prior, p(E,c), and normalizing by the plausibility, p(CC,c). The function p(CC|E,c) represents the probability of a specific pattern of activity of cortical columns, given E in the context c. A common assumption is that neural activity can be described as a Poisson-like function [22]. The term p(E,c) is the probability of E in the context c before the action of the cortical columns. In general, the prior probability reflects the frequency of E. A striking example of a cognitive influence of p(E,c) in color perception is the demonstration that prior knowledge regarding the color of objects can make achromatic images to appear as colored [23]. p(CC,c) represents the probability of a particular pattern of activity of cortical neurons.

Evidently, the brain is not merely a reactive organ. It is in large part a predictive organ [14]. Without our awareness, our brain constantly makes an enormous number of predictions of what is likely to happen next.

## 4. Neuroscience and Fuzzy Logic

**Figure 2:**
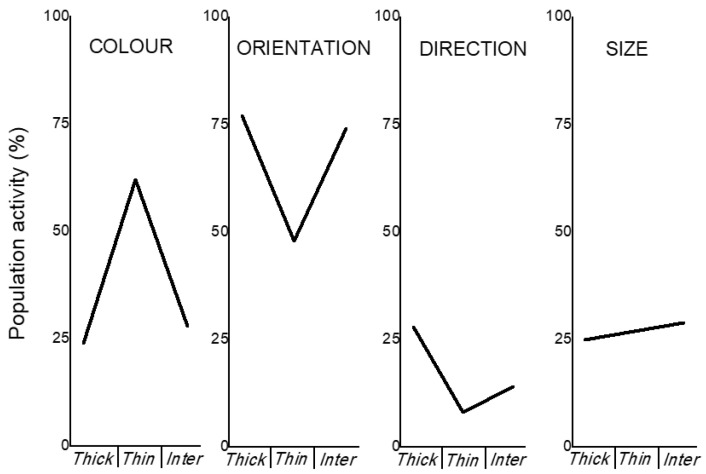
Relative population activities of neurons belonging to the thick stripes, thin stripes, and inter-stripes of the macaque monkey area V2 for color, orientation, direction of motion, and size [26].

Although the anatomical and functional columnarity of the neocortex has never been in doubt, the size, cell composition, synaptic organization, expression of signaling molecules, and function of various types of columns are dramatically different [24]. Despite these differences, cortical columns have a common functional feature. Experimental evidence has demonstrated that the functional boundaries between cortical columns are not sharp, i.e., binary, but gradual, i.e., fuzzy [25]. For instance, in the somatosensory cortex, neurons with the same cutaneous modality property, belonging to adjacent columns, are related to adjoining and overlapping peripheral receptive fields [20]. At the same time, some adjacent cortical regions map widely separated body parts, and some adjacent body parts are mapped to separated cortical zones. Furthermore, each column can play more than just one function. For instance, in the visual cortex, the area V2 that receives the columnar projections from area V1 is partitioned in thick stripes, thin stripes, and inter-stripes [25]. In these three types of compartments, color is not processed separately from other visual attributes, such as orientation, direction of motion, and size [26,27], as shown by the data of neural activity reported in Figure 2. It is evident that our experience of color, shape, orientation, and movement depends on the activity of neurons belonging to distinct cortical areas.

We would have had each attribute processed in only a single compartment if there was complete anatomical segregation. If there were no segregation at all, the trends of the population activities would be flat across the stripe compartments. The behavior depicted in Figure 2 is fuzzy as the stripes vary continuously in their degree of tuning. Such fuzzy behavior has also been found for orientation and color in the V1 cortical area, and it is maintained in V4, receiving output from thin stripes and inter-stripes, and V3 and V5, receiving output from thick stripes [26,27,28]. Cerebral events, such as color perception, partition cortical columns in fuzzy sets.

**Figure 3:**
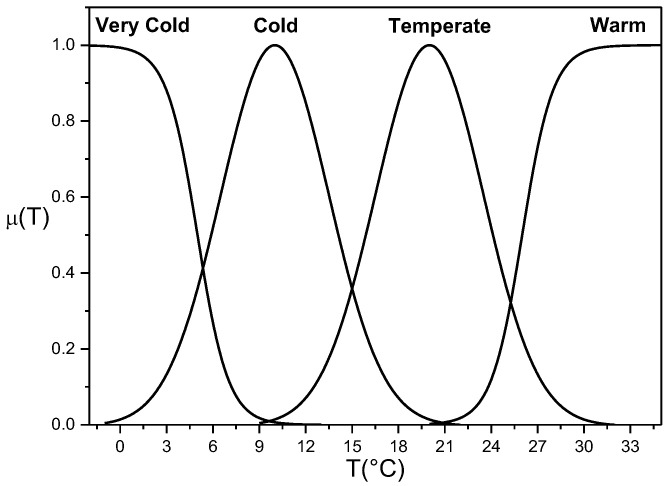
Granulation of the variable T (whose values are expressed in Celsius degrees) in four fuzzy sets that are labelled by the adjectives written on top of them.

A fuzzy set, proposed for the first time by the engineer Lotfi Zadeh in 1965 [29], is different from a classical Boolean set, as it breaks the Law of Excluded Middle. The Law of Excluded Middle states that an element y belongs to either set S or to its complement, i.e., set not-S. A Fuzzy set S of a universe of discourse U is defined by a membership function $\mu_{S}:U\to \left[0,1\right]$, which associates with each element y of U a number $\mu_{S}\left(y\right)$ in the interval [0,1]. $\mu_{S}\left(y\right)$ represents the grade of membership of y in S. Fuzzy set can wholly include ($\mu_{S}\left(y\right)=1$) or wholly exclude ($\mu_{S}\left(y\right)=0$) elements, but it can also partially include and exclude other elements ($0<\mu_{S}\left(y\right)<1$). An element y may belong to both set S and its complement not-S. An element y may belong to any set, but with different degrees of membership. The membership function $\mu_{S}$ can assume an unlimited number of shapes: it can be Gaussian, sigmoidal, triangular, or trapezoidal, just to cite a few examples. Fuzzy sets are the indispensable elements for processing fuzzy logic. Fuzzy logic has been defined as the rigorous logic of vague reasoning [30]. It allows one to describe any non-linear cause and effect relationship among input and output variables through syllogistic statements of the type “If…, Then…” involving the natural language. For any nonlinear cause and effect relationship, a Fuzzy Logic System (FLS) must be built. The construction of an FLS requires three fundamental steps, if the Mamdani’s method is used [31]. In the first step, every variable is partitioned in a certain number of Fuzzy sets. For instance, Figure 3 shows the partition or “granulation” of the variable temperature (T) into four fuzzy sets having distinct shapes and positions along the T axis. The number, shape, and position of the fuzzy sets are context-dependent. In the second step, each fuzzy set is labelled by a linguistic variable (often an adjective). This operation is called “graduation” of the variable (see the adjectives labeling the fuzzy sets in Figure 3).

**Figure 4:**
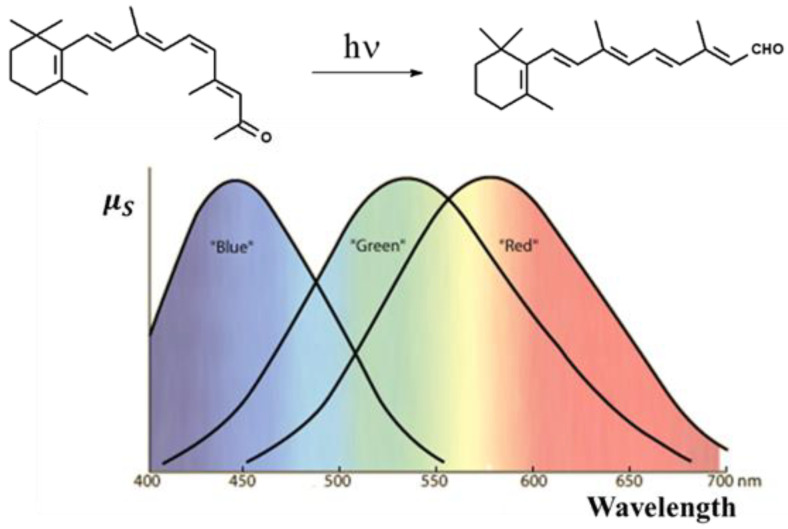
Absorption spectra of the cones (“Blue”, “Green”, and “Red”) that partition the spectral region of visible light in three Fuzzy sets. The chromophore responsible for the bands is always the retinal that photo-isomerizes from 11-*cis* to all-*trans* configuration after absorption of a photon having the right frequency.

In the third step, the causal relationships between the input and output variables are expressed through syllogistic statements of the type “If…, Then…” The antecedent, i.e., the “If…” part, contains the words labelling the input fuzzy sets. On the other hand, the consequence, i.e., the “Then…” part, includes the words labelling the output fuzzy sets. If the fuzzy rules are based on multiple inputs, the input fuzzy sets are connected through the AND, OR, and NOT operators [31]. AND corresponds to the intersection (e.g., the intersection of two fuzzy sets, whose membership functions are $\mu_{S_{1}}$ and $\mu_{S_{2}}$, can be $\mu_{S_{1}\cap S_{2}}=\min\left[\mu_{S_{1}},\mu_{S_{2}}\right]$ or $\mu_{S_{1}\cap S_{2}}=\mu_{S_{1}}\times \mu_{S_{2}}$); OR corresponds to the union (e.g., the union of the two sets S_1_ and S_2_ can be $\mu_{S_{1}\cup S_{2}}=\max\left[\mu_{S_{1}},\mu_{S_{2}}\right]$ or $\mu_{S_{1}\cup S_{2}}=\mu_{S_{1}}+\mu_{S_{2}}-\mu_{S_{1}}\times \mu_{S_{2}}$); and NOT corresponds to the complement (e.g., the membership function for the fuzzy complement of S is $\mu_{\bar{S}}=1-\mu_{s}$). Fuzzy rules may be provided by experts or can be extracted from numerical data when methods alternative to the Mamdani’s one, are selected [31]. After the three steps procedure, we have an FLS wherein we distinguish three components: the Fuzzifier, based on the partition of the input variables in fuzzy sets, and transforming numerical values of the inputs in vectors of degrees of membership to the input fuzzy sets; the Fuzzy Inference Engine that contains the “If…, Then…” rules linking input fuzzy sets to output fuzzy sets; the Defuzzifier, transforming the activated output fuzzy sets in discrete outputs. These are only the primary features of fuzzy logic that models the human capability of computing with words and thoughts [30,32]. Computing with words and thoughts allows humans to perform a wide variety of physical and mental tasks without any quantitative measurements and any numerical computations. Everyday examples of such tasks are parking a car, playing sports, deciphering sloppy handwriting, and summarizing a story. These performances are based on the brain’s crucial ability to reason with perceptions of variables such as time, distance, speed, force, direction, shape, intent, truth, possibility, likelihood, and other attributes of physical and mental objects. The perceptions of all these variables are labelled through words [33]. The power of fuzzy logic to model human capability to compute with words is partly attributable to some structural and functional analogies between a Fuzzy Logic System and the human nervous system [34]. The analogies between fuzzy logic and the human sensory system have been already evidenced in previous works [34,35] and are briefly summarized hereinafter. The sensory system is a collection of Fuzzifiers: they are the visual, auditory, tactile, proprioceptive, nociceptive, thermo-receptive, olfactory, and gustatory systems. As an example, in the visual system, the three types of photoreceptor proteins, belonging to the three types of cones, “Blue”, “Green”, and “Red”, allow humans to distinguish colors. They have three absorption spectra that look similar to three fuzzy sets “granulating” the visible spectrum [34] (see Figure 4). Lights, having distinct spectral compositions (or the so-called modalities), belong to the three spectral fuzzy sets at different degrees. The modality of a light stimulus is encoded as degree of membership of the light to the three “Molecular Fuzzy sets” (i.e., $\mu_{Blue}\left(light\right),\;\mu_{Green}\left(light\right),\;\mu_{Red}\left(light\right)$) that are the three absorption spectra shown in Figure 4. Furthermore, each cone contains millions of replicas of one particular photoreceptor protein. Therefore, light stimuli, having the same spectral composition but different intensities, excite different amounts of photoreceptor proteins within each cone. In other words, each cone looks similar to a “Cellular Fuzzy set”, and lights differing just in the intensity belong to this “Cellular Fuzzy sets” at different degrees. Whereas the hue of a color is encoded at the molecular level, its saturation and brightness are encoded at the cellular level [34]. Finally, the spatial distribution of a visual signal is encoded at the tissue level, through the array of sensory cells covering the retina.

The Fuzzy information collected by the sensory cells is processed further by the afferent neurons, whose receptive fields are similar to fuzzy sets encompassing collections of the photoreceptor cells [35]. Finally, the information is processed into the cortex and, as demonstrated in this work, the cortical columns work as fuzzy sets as different cerebral events, such as the integration of the signals coming from distinct senses or receptive fields of every sensory organ, belong to the cortical columns at different degrees. The brain as a whole appears to have a highly distributed functionality with many different areas making important contributions to every its function [36]. The perception-based information and its elaboration are vague and fuzzy as the human nervous system appears to be fuzzy at structural and functional levels.

## 5. Fuzzy Logic and Bayesian Probability

**Figure 5:**
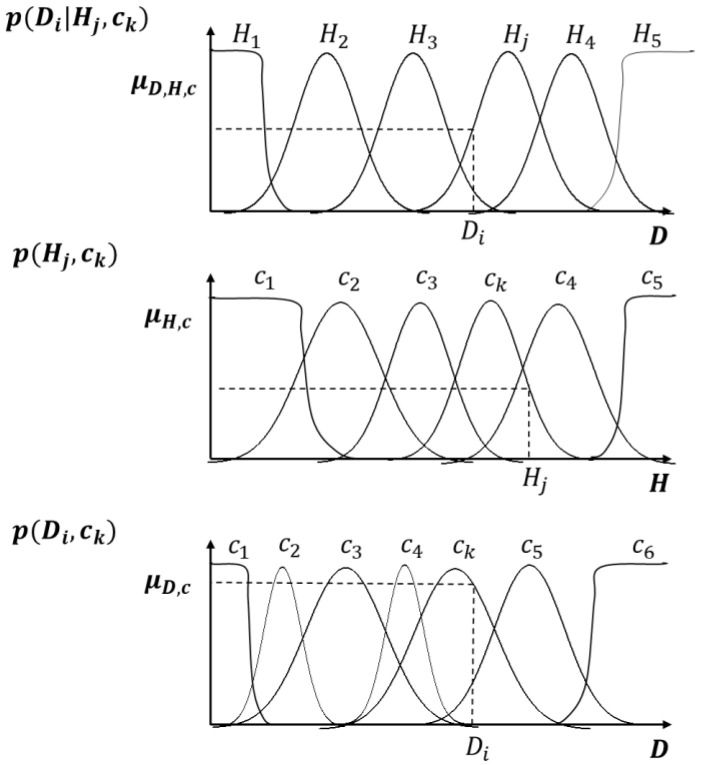
Representations of the information contained in the likelihood (top panel), prior probability (middle panel), and plausibility (bottom panel) in terms of fuzzy sets theory.

The relation between the theory of fuzzy sets and the probability theory has been debated for a long time [31]. Fuzzy sets theory was born to describe partial truths, i.e., degrees of truths. According to the “Principle of Valence”, proposed by Lukasiewicz at the beginning of the twentieth century, “every proposition has a truth-value” [37]. For Lukasiewicz, propositions are not only either true or false, according to the “Principle of Bivalence”, formulated by Chrysippus and his school in ancient Greece, but they can have an intermediate truth-value. Truth-values are modeled by numbers in the unit interval. Fuzzy sets theory embodies the “Principle of Valence”. It derives that fuzzy logic is a logic of partial degrees of truth, imprecise notions, and propositions, which may be more or less true, and expressed through the natural language. On the other hand, probability theories have been developed to describe the degrees of uncertainty when there is lack of knowledge about the truth. Fuzzy sets theory has embraced uncertainty modeling when membership functions have been reinterpreted as possibility distributions [38,39]. This conceptual extension was necessary when fuzzy logic was used in pattern recognition and control engineering [40]. Possibility theory is an uncertainty theory devoted to the handling of incomplete information [41]. Zadeh articulated the relationship between possibility and probability, noticing that what is probable must preliminarily be possible [38]. The possibility distribution function π represents the state of knowledge of an agent about the actual state of affairs, distinguishing what is more plausible from what is less plausible, what is the normal course of things from what is not, what is surprising from what is expected. The function π will be 0 in case a state is impossible, whereas it will be 1 when the state is totally possible. All the other situations are represented by all the real numbers included between 0 and 1. The possibility distribution function has been identified with the likelihood function of the Bayes’ formula [42,43,44]. According to the Bayes’ formula, the combination of the current information, represented by the likelihood, with past information, embodied in the prior probability, allows one to make a prediction about a possible but uncertain future event by calculating the posterior probability [45]. Information, as properly evidenced by Zadeh, is restriction [11]. A restriction on a variable X, R(X), is a limitation on the values that X can take. The restriction on X is an answer to the question: What is the value of X? A restriction is singular if R(X) is a singleton (i.e., a number). A restriction is nonsingular if R(X) is not a singleton. Non-singularity implies uncertainty. Any non-singular restriction, expressed either through a word of natural language or an interval of numerical values of the type R(X)=x±ε, with ε being the error associated with a scientific measurement, can be interpreted as a fuzzy set. The likelihood p(D|H,c) is information based on the granulation of all the data D in fuzzy sets that are all the reasonable hypotheses H that can be formulated in the context c. Therefore, $p(D_{i}|H_{j},c_{k})$ will be the degree of membership $\mu_{D,H,c}$ of $D_{i}$ to the $H_{j}$ fuzzy set, defined in the $c_{k}$ context (see the top panel of Figure 5). The prior probability p(H,c) is information rooted in the partition of all the possible hypotheses in fuzzy sets that are the contexts. Hence, $p\left(H_{j},c_{k}\right)$ will be the degree of membership $\mu_{H,c}$ of $H_{j}$ hypothesis to the $c_{k}$ fuzzy set (see the middle panel of Figure 5). Finally, the plausibility p(D,c) is based on the granulation of the data D in fuzzy sets that are the contexts, and $p\left(D_{i},c_{k}\right)$ will represent the degree of membership $\mu_{D,c}$ of $D_{i}$ to the $c_{k}$ fuzzy set (see the bottom panel of Figure 5).

Based on this interpretation, the Bayes’ formula becomes a fuzzy inference wherein the posterior probability is the intersection between the prior and likelihood, implemented through the product $p\left(D|H,c\right)\cdot p\left(H,c\right)=\left(\mu_{D,H,c}\cdot \mu_{H,c}\right)$, and normalized by the plausibility ($p\left(D,c\right)=\mu_{D,c}$).

This interpretation of the Bayes’ formula can be extended to the case of cortical columns CC and the cerebral event E appearing in Equation (1). It derives that the cortical columns CC are granulated in Fuzzy sets by the cerebral event E, and the likelihood p(CC|E,c) represents the degree of membership of the cortical columns to E in the context c. The prior probability represents the degree of membership of E to the context c as different contexts granulate the cerebral events in Fuzzy sets. The contexts also partition the cortical columns in fuzzy sets, and the plausibility p(CC,c) is the degree of membership of CC to c. The posterior probability p(E|CC,c) is the prediction that is possible to be made after intersecting (i.e., multiplying) the present cerebral information p(CC|E,c) [18] with the memorized past information p(E,c) (see Equation (1)) normalized by p(CC,c).

## 6. Quantum Mechanics and Bayesian Probability: The QBism

Quantum mechanics is that scientific theory that tries to describe any natural phenomenon from the point of view of atoms and subatomic particles. It was first formulated by Bohr and Rutherford at the beginning of the twentieth century [46] after experiencing strange phenomena which could not be explained by classical physics. Quantum mechanics asserts that there are experiments for which the exact outcome is fundamentally unpredictable. In these cases, one has to be satisfied with computing the probabilities of the various outcomes [47]. Quantum theory results effective in interpreting not only microscopic events but also specific macroscopic phenomena, such as superfluid helium, superconductors, laser light, and Bose–Einstein condensates [48].

In quantum mechanics, the state of a system is described by a vector |ψ〉 with norm equal to 1 (〈ψ|ψ〉=1) defined in an appropriate Hilbert’s complex space. The elementary unit of quantum information is the qubit (|φ〉), which is a superposition of two states, $|\psi_{1}\rangle$ and $|\psi_{2}\rangle$:

(2) $$\left|\varphi \rangle =\alpha \right|\psi_{1}\rangle +\beta |\psi_{2}\rangle$$

with α and β being two complex numbers, which verify the normalization condition: ${\left|\alpha \right|}^{2}+{\left|\beta \right|}^{2}=1$. When there is high uncertainty on the state of a system and many different wavefunctions $|\psi_{i}\rangle$ are possible, the state of a system is represented by a matrix ρ defined as:

(3) $$\rho =\sum_{i}p_{i}|\psi_{i}\rangle \langle \psi_{i}|$$

It is a mixed state with each $|\psi_{i}\rangle$ having probability $p_{i}$. The matrix ρ has unitary trace: Tr[ρ]=1.

All the various interpretations of quantum mechanics see quantum states as physical entities, such as a blob of ψ-flavored gelatin, sliding about in accord with its own dynamical laws [49]. A peculiar interpretation is offered by QBism that is grounded on an epistemic view of quantum states [50]. The guidance towards QBism started in the early 1990s under the influence of what Edwin James said in 1980s: The present quantum mechanical formalism is “a peculiar mixture describing in part realities of Nature, in part incomplete human information about Nature—all scrambled up by Heisenberg and Bohr into an omelette that nobody has seen how to unscramble. Yet, we think that the unscrambling is a prerequisite for any further advance in basic physical theory. For, if we cannot separate the subjective and objective aspects of the formalism, we cannot know what we are talking about; it is just that simple” [51]. Hence, quantum mechanics is a rather ill-defined mixture of both epistemic and ontic elements. According to the QBism, quantum states fall in the epistemic spectrum of: (1) Information; (2) Epistemic knowledge; (3) Belief (doxastic notion); and (4) Pragmatic Gambling Commitments (Opportunistic formulation of quantum states) [52]. QBism excludes an ontic interpretation of quantum states. The first tenet of QBism states that all quantum probabilities, including probability-1 assignments, are so personal or subjective they never tell nature what to do. Quantum states have no “ontic hold” on the world. Quantum probabilities are Bayesian probabilities as they are numerical measures of personal degrees of belief [8]. According to QBism, quantum mechanics is “a tool anyone can use to evaluate, on the basis of one’s past experience, one’s probabilistic expectations for one’s subsequent experience” [53]. The collection of data on quantum systems requires performing experiments. The second tenet of QBism declares that a quantum measurement is any action an agent takes upon the world, and its outcome is the agent’s resulting experience and therefore personal to the agent who performs the measurement action. In every quantum measurement set by an experimenter’s free will, the world is shaped just a little as it takes part in a moment of creation. Quantum measurements represent those moments of creation that are sought out or noticed [54]. According to the third tenet of QBism, a measurement apparatus is conceptually an extension of the agent. It should be considered analogous to a prosthetic sensory organ, i.e., simultaneously a tool and a portion of the individual. In a quantum measurement, the world is split into two pieces: one piece is the gambling agent and the other piece is the object of enquiry. The quantum state |ψ〉 represents the agent’s expectation about the outcome of the experiment. A quantum state determines probabilities through the Born rule. According to the fourth tenet of QBism, the Born rule is a normative statement. It has objective character as it is about the decision-making behavior any individual agent should strive for. First, a Positive Operator-Valued Measure (POVM) can be associated to any facility. A POVM is a set $\left\{\Pi_{0},\Pi_{1},\;\Pi_{2},\;\ldots \right\}$ of positive operators $\Pi_{k}$, whose sum is equal to the identity: $\underset{k}{\sum }\Pi_{k}=I$. Every possible outcome $l_{k}$ of the measurement is linked to an element $\Pi_{k}$ of the POVM $\left\{\Pi_{0},\Pi_{1},\;\Pi_{2},\;\ldots \right\}$. When the state of the quantum system is ρ, the probability $p_{k}$ of obtaining the *k*-th result is given by

(4) $$p_{k}=Tr\left[\rho \Pi_{k}\right]\;$$

This equation is known as Born’s rule. If the quantum system is in a pure state |ψ〉, the Born’s rule becomes: $p_{k}=\langle \psi \left|\Pi_{k}\right|\psi \rangle$. The positivity of the POVM’s elements guarantees that all the $p_{k}$ values are non-negative. The condition $\underset{k}{\sum }\Pi_{k}=I$ is required to assure that $\underset{k}{\sum }p_{k}=1$. The QBism proposes a formalism that can ignore quantum states entirely and only use probabilities. The key ingredient is a structure called “Symmetric Informationally Complete Positive-Operator-Valued Measure” (SIC-POVM). It is a set of $d^{2}$ rank-one projection operators $\left\{R_{0},R_{1},\;\ldots ,\;R_{i},R_{j},\;\ldots \right\}$ on a finite *d*-dimensional Hilbert space. These operators are not orthogonal to each other, but have a very special symmetry that if we take the inner product of any two of them, its trace is equal to a constant value given by

(5) $$tr\left[R_{i}R_{j}\right]=\frac{1}{d+1}$$

**Figure 6:**
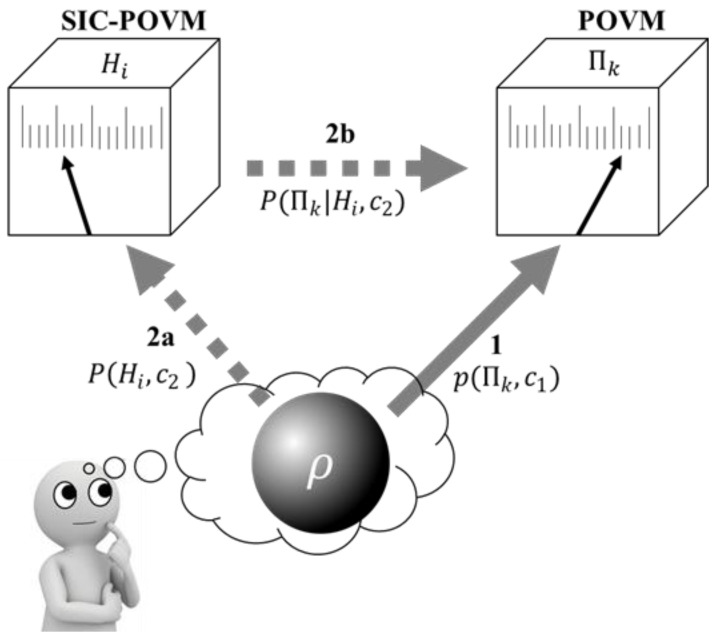
Schematic representation of the two alternative paths (1 and 2) for measuring the quantum state ρ.

Equation (5) (wherein i≠j) means that there is a constant overlap between any two projection operators or, in other words, the SIC-POVM is a set of $d^{2}$ lines in a complex vector space such that the angle between any two of them is the same. This set is called a maximal set of equiangular lines. If it exists, then there exists a way of re-expressing quantum states as linear combinations of these projections:

(6) $$\rho =\sum_{i=1}^{d^{2}}\left[\left(d+1\right)p(R_{i})-\frac{1}{d}\right]R_{i}$$

with $p(R_{i})=\frac{1}{d}tr\left[\rho R_{i}\right]$. The $R_{i}$ are linearly independent and $\overset{d^{2}}{\underset{i=1}{\sum }}\left(\frac{1}{d}R_{i}\right)=I$ [55]. A SIC-POVM gives a whole different way to think of the Born rule [56]. As depicted in Figure 6, any quantum measurement for a given quantum system ρ can be conceptualized in two ways. The first way is in the context ($c_{1}$) of any standard von Neumann measurement whose probability assignment is expressed by Equation (4), which is rewritten in the following format:

(7) $$p\left(\Pi_{k},c_{1}\right)=Tr\left[\rho \Pi_{k}\right]$$

The second way is in a context ($c_{2}$) that entails a SIC-POVM, followed by a standard von Neumann measurement. If $P\left(H_{i},c_{2}\right)=d*p(R_{i})$ represents the agent’s personal probabilities for the outcomes of the SIC-POVMs, and $P(\Pi_{k}|H_{i},c_{2})$ is his conditional probability for the outcomes of the following von Neumann measurement, then the Bayesian definition of total probability allows one to define the probability $P\left(\Pi_{k},c_{2}\right)$ the agent would assign to the POVM measurement:

(8) $$P\left(\Pi_{k},c_{2}\right)=\sum_{i=1}^{d^{2}}P\left(H_{i},c_{2}\right)P(\Pi_{k}|H_{i},c_{2})$$

The outputs of the two measurement paths are different: $p\left(\Pi_{k},c_{1}\right)\neq P\left(\Pi_{k},c_{2}\right)$. However, $p\left(\Pi_{k},c_{1}\right)$ is still a function of $P\left(\Pi_{k},c_{2}\right)$. In fact, it results that

(9) $$p\left(\Pi_{k},c_{1}\right)=\left(d+1\right)P\left(\Pi_{k},c_{2}\right)-1=\left(d+1\right)\sum_{i=1}^{d^{2}}P\left(H_{i},c_{2}\right)P(\Pi_{k}|H_{i},c_{2})-1$$

It derives that the Born rule is nothing but a kind of quantum law of total probability. It is an extension of Bayesian probability as it gives extra normative rules to guide the agent’s behavior when the agent interacts with the physical world [50]. This kind of extension has been shown to be indispensable to describe how certain biosystems process information and make decisions: classical probability is unsatisfactory; quantum probability is required [57,58,59].

Hence, QBism cancels out wavefunctions: quantum mechanics is couched directly in terms of probabilities, that is, real numbers between 0 and 1, by passing wavefunctions with their nebulous status and imaginary components. QBism views quantum theory as an addition to decision theory. Probabilities are Bayesian, i.e., epistemic as they are logical constructs rather than physical realities and in which probability statements do apply directly to individual events [60]. Quantum mechanics describes what an observer (the subject) experiences while contemplating nature (the object). In 1961, Bohr said: “Physics is to be regarded not so much as the study of something a priori given, but as the development of methods for ordering and surveying human experience”. By the “a priori given”, Bohr meant the external world—what Einstein called “reality.” According to QBism, probability does not exist. The Bayesian probability should be regarded as a degree of belief and thereby dependent on an agent’s experience. By switching from frequentist to Bayesian probability, QBism injects human thoughts and beliefs into the austere mathematical framework of physics. Quantum mechanics, according to QBism, is not a description of the world, but a technique for comprehending it. QBism puts the scientist back into science [61]. QBism represents a radical narrowing as it restricts the relevance of a probability estimate to a single agent. However, at the same time, it represents an immense broadening, as included among the experiences of that agent are all their personal experiences, past and present. Louis Menand, in his book “Metaphysical Club: A Story of Ideas in America” [62], talks about the U.S. Supreme Court Justice Oliver Wendell Holmes, Jr., who used to say that “Complete certainty is an illusion; of that he was certain. There were only greater and lesser degrees of certainty… We cannot know what consequences the universe will attach to our choices, but we can bet on them, and we do it every day.” Holmes liked to call himself a “bettabilitarian”, and, consequently, QBism has been renamed as Quantum Bettabilitarianism [49]. Although any agent has considerable freedom to assign probability estimates to their own future experiences, they must conform to the restrictions of the calculus of probabilities.

## 7. Quantum Mechanics and Fuzzy Logic

The quantum mechanics principle of superposition of states finds a counterpart in the degree of membership to fuzzy sets [63,64,65]. However, the mathematical frameworks for the two concepts are different [66,67]: fuzzy logic is based on t-norms and t-conorms algebras for intersection and union, respectively, on membership values of fuzzy sets, while quantum mechanics is based on the Hilbert space formalism. Nevertheless, there are noteworthy attempts at bridging quantum with fuzzy logic [68]

An attempt exploits the possibility of having a qubit evolving on a unit circle in the R^2^ plane. The topological equivalence of a circle and a square allows replacing the coefficients of the qubit by a pair of membership functions, which evolve on the perimeter of the unit square [66]. In practice, the qubit can be then implemented by storing the two membership functions in a pair of fuzzy memory elements such as fuzzy flip-flops [69].

Another attempt is based on the formulation of complex fuzzy sets [70], i.e., fuzzy sets characterized by complex-valued membership functions, whose range is not limited to [0,1] but extended to the unit circle in the complex plane. The use of a complex value associates “wave-like” qualities with membership functions, which become similar to quantum wavefunctions. Complex membership functions can interfere, constructively and destructively, in a similar manner to waves.

The interpretation of the quantum mechanics according to the QBism allows the establishment of a new link between quantum and fuzzy logic. According to QBism, there is no unique wavefunction. Wavefunctions are assigned by an agent and depend on the total information available to the agent. They do not exist in nature [71]; they are subjective and malleable. Wavefunctions and measurement operators are viewed epistemically. Both of them are similar to fuzzy sets. Position, energy, speed, direction of motion, and many other attributes of a quantum particle can be spread out in the wavefunction over different possibilities until a measurement is made and a single value is unambiguously picked out. The collapse of a wavefunction is not a physical event, i.e., a change in the state of the system triggered by an experiment. It is rather a Bayesian updating of a probability assignment upon the acquisition of new information [14]. Any measurement on a quantum system can also be interpreted as a fuzzy inference. Wavefunctions are fuzzy sets granulating the quantum system’s features. A POVM is a collection of fuzzy sets that are the positive operators $\left\{\Pi_{0},\Pi_{1},{\;\Pi }_{2},\;\ldots \right\}$. The Born rule (Equation (4)) represents the defuzzification event that transforms the intersection of the wavefunction- and measurement operator-fuzzy sets in a discrete value. Such discrete value constitutes a prediction for the outcome of the actual experiment. As John Archibald Wheeler declared, “Each elementary quantum phenomenon is an elementary act of creation” [72] as the collected data allow to update the subjective information about the quantum system. It derives that quantum theory is a universal single-user theory in much the same way that Bayesian probability theory and fuzzy logic are. For any Qbist, quantum theory is not something outside probability theory, but an addition to probability theory itself. The introduction of the SIC-POVM allows the formulation of a new equation for predicting the outcomes of the measurements. Equation (9) is an extension of the Bayesian probability theory and if we conceive the SIC-POVM a set of $d^{2}$ fuzzy sets, it becomes a new algorithm for running a fuzzy inference engine and performing the defuzzification.

The attempts to establish connections between quantum and fuzzy logic are worth pursuing as they could unveil new strategies for implementing the intrinsic parallelism of quantum computation.

## 8. Perspectives

This paper establishes a new link between fuzzy logic, neuroscience, and quantum mechanics through Bayesian probability theory. According to the Bayes’ theory, probabilities are personal beliefs that an event will occur or a hypothesis will be true. Any belief or posterior probability is a function of the product between the prior probability, depending on past experiences, and the likelihood, which grounds on the present data. This product is normalized by the plausibility (see Figure 1). All these terms of the Bayes’ formula are implemented in our brain through the spatiotemporal patterns of activity of cortical columns in the neocortex. Priors are probably formed through spontaneous brain dynamics during resting periods across both cortical and subcortical structures [73]. Experimental evidence has shown that cortical columns behave as fuzzy sets (as shown in paragraph 4). Hence, the terms of Bayes’ formula are describable as degrees of membership to fuzzy sets, and the Bayesian inference is conceivable as a fuzzy inference (as claimed in paragraph 5). A kind of fuzzy inference is also the Born rule if the probabilities that appear in quantum mechanics are interpreted as Bayesian probabilities, in agreement with the QBism’s point of view (see paragraphs 6 and 7). In quantum mechanics, both the wavefunctions and the positive operators associated with any POVM and SIC-POVM are conceivable as fuzzy sets.

The fuzzy interpretation of Bayesian inference incites to always devise a new strategy to process fuzzy logic. Fuzzy logic is routinely implemented in digital electronic devices. Analog electronic circuits are more appropriate for building Fuzzy Logic Systems as the electrical signals do not change abruptly in a sigmoid manner but smoothly in a hyperbolic or linear way [64]. More recently, molecules are drawn attention as promising tools for processing fuzzy logic [10].

When a molecular compound exists in many conformers and/or it experiences distinct micro-environments, it is describable as a quantum mixed state:

(10) $$\rho =\sum_{i=1}^{N}w_{i}|\psi_{i}\rangle \langle \psi_{i}|$$

In Equation (10), $w_{i}$ represents the weight of the *i*-th wavefunction and corresponds to its probability $p_{i}$ appearing in Equation (3). The term $w_{i}$ is also the fuzzy unit of information. The molecular system represented by ρ will have a Fuzzy Entropy, H [74] given by:

(11) $$H=-\frac{1}{logN}\sum_{i=1}^{N}w_{i}log\left(w_{i}\right)$$

H assumes any real value included between 0 and 1. If the compound exists in just one state (i.e., one conformer experiencing just one micro-environment), N=1, $w_{i}=1$, and H=0. On the other hand, if the compound exists in N distinct states (as it has N conformers or it experiences N different micro-environment), which are equally probable, then $w_{i}=1/N$ and H=1. Of course, there is an infinite number of other possibilities which originate H values included between 0 and 1. The ensemble of N states constitute a molecular fuzzy set.

**Figure 7:**
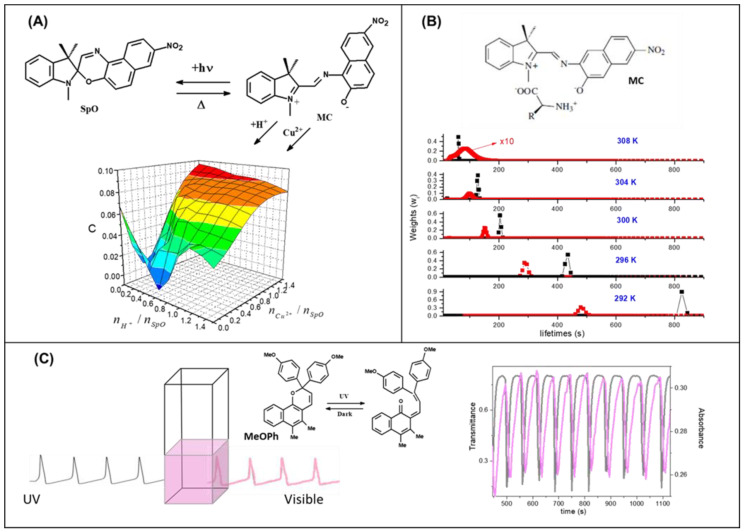
Smooth analog dependence of the SpO’s colorability on the chemical inputs H^+^ and Cu^2+^, in (**A**) [78]. Lifetimes’ distributions for the merocyanine MC as a function of temperature and with (black points) and without (red points) the ammino-acid, in (**B**) [75]. Periodic variation of color’s saturation for the naphthopyran MeOPh receiving a periodic UV signal, in (**C**) [86].

It is possible to process fuzzy logic when physical or chemical inputs modify the $w_{i}$ values, and hence H (see Figure 7B) [75]. The estimation of the $w_{i}$ values appearing in Equations (10) and (11) can be accomplished by recoding spectroscopic time-resolved signals and fitting any transient signal through the Maximum Entropy Method (MEM) [76]. For instance, MEM fits a fluorescent decay signal ($I_{em}$) by using a poly-exponential function with N terms [77]:

(12) $$I_{em}=\sum_{i=1}^{N}w_{i}e^{-\frac{t}{\tau_{i}}}$$

It is possible to process fuzzy logic even though time-resolved techniques are not available. What is required is to find smooth analog inputs-outputs relationships (see Figure 7A) that can employed to implement Fuzzy Logic Systems after the granulation and graduation of all the variables and the formulation of fuzzy rules [78,79,80,81,82,83].

The granulation of the physicochemical variables can be accomplished not only a posteriori through software but also a priori by mixing proper molecular compounds and imitating the structural principle of every human sensory subsystem. For instance, in paragraph 4, we have evidenced the three retinals we have in the cones partition the visible spectral region in three fuzzy sets. This molecular granulation of the visible region confers humans the power of distinguishing colors. Such an approach has been applied in the design of a system with three or more photochromic compounds, which has allowed us to extend human vision to the UV region [84,85].

Finally, the chemical systems, which mimic neural dynamics, communicate through optical signals, and give rise to synchronization phenomena [86,87], might be applied to implement complex fuzzy sets [70]. For instance, a photochromic compound that receives an excitatory periodic UV signal coming from another artificial neuron model gives rise to a color whose saturation oscillates (see Figure 7C) [88,89]. The values of the RGB color coordinates become the real part of the complex membership function, whereas their periodic variation represents its imaginary part.

The parallelism of fuzzy logic based on the co-existence of different fuzzy sets is promising for developing an artificial intelligence that is said to be chemical as it has roots in molecular performances [90]. Chemical Artificial Intelligence (CAI) draws inspiration from the structure and performances of the human nervous system and tries to approach Bayesian inference.

Another challenge is to devise new algorithms that exploit fuzzy information. The Born rule, as expressed through Equation (9) by QBism, becomes a new method for calculating probabilities and also a new fuzzy inference rule. This extension of the probability theory will probably allow strengthening the relationship between fuzzy logic and quantum mechanics. If so, it will contribute to boosting unconventional computing [91] for facing Computational Complexity.

## Data Availability

Not applicable.
